# A method for predicting individual residue contributions to enzyme specificity and binding-site energies, and its application to MTH1

**DOI:** 10.1007/s00894-016-3119-5

**Published:** 2016-10-06

**Authors:** James J. P. Stewart

**Affiliations:** Stewart Computational Chemistry, 15210 Paddington Circle, Colorado Springs, CO 80921 USA

**Keywords:** PM7, Noncovalent interactions, Docking, Binding, Enzyme specificity, MTH1, Nucleotide hydrolysis

## Abstract

A new method for predicting the energy contributions to substrate binding and to specificity has been developed. Conventional global optimization methods do not permit the subtle effects responsible for these properties to be modeled with sufficient precision to allow confidence to be placed in the results, but by making simple alterations to the model, the precisions of the various energies involved can be improved from about ±2 kcal mol^−1^ to ±0.1 kcal mol^−1^. This technique was applied to the oxidized nucleotide pyrophosphohydrolase enzyme MTH1. MTH1 is unusual in that the binding and reaction sites are well separated—an advantage from a computational chemistry perspective, as it allows the energetics involved in docking to be modeled without the need to consider any issues relating to reaction mechanisms. In this study, two types of energy terms were investigated: the noncovalent interactions between the binding site and the substrate, and those responsible for discriminating between the oxidized nucleotide 8-oxo-dGTP and the normal dGTP. Both of these were investigated using the semiempirical method PM7 in the program MOPAC. The contributions of the individual residues to both the binding energy and the specificity of MTH1 were calculated by simulating the effect of mutations. Where comparisons were possible, all calculated results were in agreement with experimental observations. This technique provides fresh insight into the binding mechanism that enzymes use for discriminating between possible substrates.

## Introduction

### Background

Factual knowledge of how enzymes catalyze reactions comes from several sources, of which the more important are biochemical experimentation, X-ray analysis, and NMR analysis. In recent years these sources of data have been augmented by the development of computational chemistry modeling tools that can be used for investigating and understanding protein–ligand interactions (for reviews, see [1, 2]).

In recent years, the semiempirical method PM7 [3] has also been shown to be useful for detecting errors in the X-ray structures of proteins [4], removing some of these errors [5], and exploring the applicability of these methods to the modeling of the entire MTH1 enzyme [6], a system within the Protein Data Bank [7] (PDB) file 3ZR0. With the exception of a single fault where some noncovalent contact distances were shorter than those reported in the PDB file, PM7 has been shown to reproduce, both qualitatively and quantitatively, many of the structural features of enzymes.

Before any method, either experimental or theoretical, can be regarded as useful, it must be shown to provide information that provides an insight that cannot be obtained at all or as easily using other techniques. In the case of a substrate docking into a binding site in an enzyme, X-ray and NMR analysis allow the geometries of binding sites to be examined, and biochemical experiments can be used to determine the significance and roles of individual residues. With the possible exception of the POLARIS model of the program MOLARIS [8], what has not been available has been a simple method for quantifying the individual energy contributions of each residue to binding or to specificity; that is, the ability of the enzyme to discriminate between candidate substrates.

In general, chemical processes are dominated by energies. For example, the efficiency of binding of a substrate into an enzyme depends on the energies of the separated and bound systems, and on the energies involved when individual water molecules are displaced during binding. Given a computational model of a docking site, inferences could be made regarding the factors that affect the binding energy, such as the presence or absence of hydrogen bonds, charged sites, hydrophobic and other steric interactions, etc., but hitherto the direct prediction of the influence on the energy of the presence of the various moieties involved has not been practical.

The semiempirical method PM7 [3] was used in MOPAC2016 [9] to model the binding of a normal and an oxidized nucleotide in the enzyme MTH1. Energy terms associated with binding and specificity were calculated using a model that involved simulating the mutation of residues.

### Computational method

Until recently, full quantum chemical modeling of proteins has not been practical, even with very fast semiempirical methods such as PM7, because of the considerable computational effort involved. This is due, in part, to the fact that conventional matrix algebra methods scale as the third or higher power of the size of the system, and proteins are inherently large systems. However, by using a method based on localized molecular orbitals, MOZYME [10], this scaling has been reduced to about 1.5; as a result, the simulation of systems of several thousand atoms has now become routine.

The utility of this modeling technique can be illustrated by providing examples of its application to real systems. In one example, a comparison was made [5] between a set of recently published PDB structures and those predicted using PM7, and several questionable features—such as covalent bond lengths that were outside expected limits, unrealistically short hydrogen-bond lengths, and unexpected van der Waals contact distances—were identified. Detecting such features involves only a straightforward calculation, which suggests that, had this technique been available earlier, the presence of these anomalies in the PDB structures might have been avoided.

A method for generating a chemically more realistic geometry of the structure of a protein [4] was developed that combined experimental and PM7 computational chemistry results. Only small changes, on the order of 0.1 Å, in atomic positions were involved, but the effect on the calculated heat of formation Δ*H*_f_ was dramatic. In many cases, where the Δ*H*_f_ of the PDB structure was often several thousand kilocalories per mole above that of the theoretically predicted structure, if the atoms in the PDB structure were moved by an average of only 0.1 Å, then the energy difference decreased by over 80 %. Almost all of this decrease was attributed to corrections made to the PDB geometry; the contribution attributable to errors in the geometry caused by faults in the computational method has been shown to be much smaller [4]. Although errors in energies from PM7 were small, a fault that affected geometries was identified which caused unrealistically small separations between pairs of noninteracting residues. Fortunately, this particular error would not compromise the current work because of the presence of a hydrogen-bond network in the region of the binding site in MTH1 that provided a lattice of interactions between the residues.

Recently [6], the applicability of PM7 to model various phenomena such as site ionization, noncovalent interactions, and secondary structures (alpha helices, beta sheets, hairpin bends, etc.) that occur in proteins was examined using 3ZR0 as reference. Provided that the system used was correctly preconditioned by the addition of hydrogen atoms and the resulting geometry was optimized, most of the features of the PDB structure were reproduced with useful accuracy. More importantly, the model also provided a chemically useful description of the various structures involved that could be used in subsequent work for investigating specific phenomena.

In common with the earlier work, all systems were modeled using the COSMO implicit solvation method [11]. Implicit solvation is essential for correctly representing the electrostatic environment of the various moieties being modeled.

Within enzyme-binding sites, noncovalent interactions are often the most important. Generally, the most important of these are hydrogen bonds, dispersion, electrostatics, and electronic interactions. Heretofore, hydrogen bonds and dispersion energies in semiempirical methods were of low accuracy, but, following recent advances in the modeling of hydrogen bonds using semiempirical methods [12–14] and the development of Grimme’s D3 dispersion approximation [15], a large increase in accuracy has been achieved, as illustrated in a test of virtual screening tools where the PM6-D3H4X method was shown [16] to outperform several [17–22] widely used scoring functions. Electrostatic interactions, of which the most important are those that occur in salt bridges and other ionized sites, are straightforward to calculate. Electronic interactions between pairs of atoms that are not chemically bound together give rise to the formation of weak (i.e., noncovalent) bonds, the most common of these being hydrogen bonds. Energy contributions from bonds of this type are, of their nature, small, and decrease rapidly with increasing interatomic separation. As with electrostatic interactions, errors in electronic interactions arising from nonequilibrium structures are likely to be small.

### MTH1

MTH1 is a nucleotide-pool sanitizing enzyme. Reactive oxygen species convert normal nucleotides into oxidized nucleotides such as 8-oxo-2′-deoxyguanosine-5′-triphosphate, 8-oxo-dGTP; if these become incorporated into DNA they can cause mutations that in turn can result in cancers. MTH1 selectively destroys these harmful oxidized nucleotides by hydrolyzing the triphosphate group to yield a nucleoside monophosphate and a pyrophosphate ion. Experimentally, the Michaelis constant *K*_M_ for the substrate binding to the enzyme [23] indicates that MTH1 binds 8-oxo-dGTP more strongly than it does dGTP, implying that the binding site of the enzyme is the most likely source of the selectivity. Svensson et al. reported [24], in PDB file 3ZR0, the X-ray structure of MTH1 complexed with the product of hydrolysis 8-oxo-dGMP, and showed that, although the reactive site was near to the reaction site, the binding site was not near to the oxidized site but at the opposite end of the guanine group. That a large distance separated the oxidized and binding sites in the substrate raises the intriguing question of how the enzyme manages to distinguish between the various substrates, particularly given the absence of any important noncovalent interactions in the vicinity of the oxidized site. This report described in detail the various structures near to the binding site, and discussed possible mechanisms that could be used by MTH1 to discriminate between the various substrates.

MTH1 is of particular interest from a computational chemistry perspective in that there is a large amount of data both on the structure of the enzyme and substrate complex and on the catalytic behavior of the enzyme, but little is known regarding the energetics involved in the discrimination process.

The objective of this investigation therefore was to examine the energetics involved in docking substrates into the MTH1 enzyme. MTH1 catalyzes the hydrolysis of 8-oxo-dGTP and, to a lesser extent, dGTP to the monophosphate. In 3ZR0, only the product of hydrolysis, 8-oxo-dGMP, was present; thus, for convenience, only the monophosphates 8-oxo-dGMP and dGMP were used in modeling, the assumption being made that the geometries in the binding sites of both the monophosphate and the triphosphate substrates would be similar.

## Methods

### Initial structural model

**Fig. 1:**
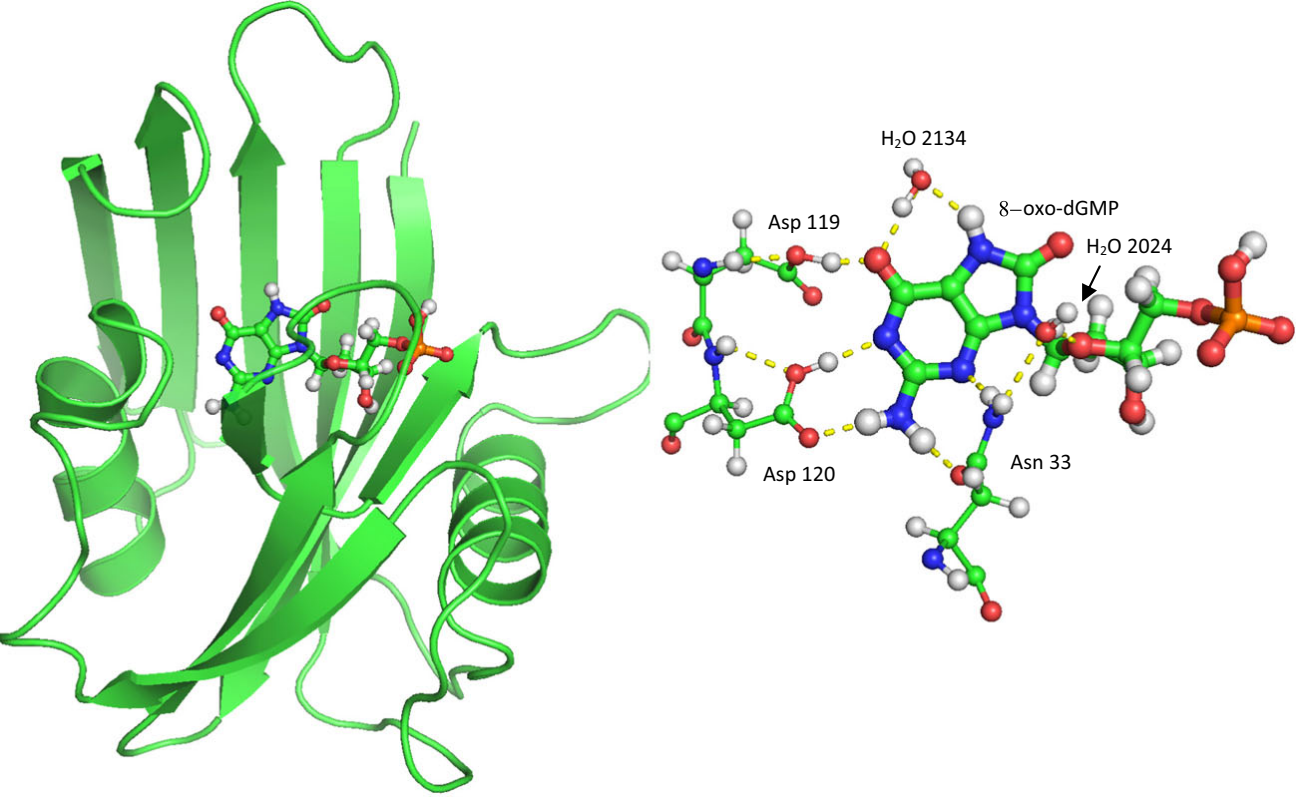
MTH1 plus 8-oxo-dGMP substrate, showing the substrate in the binding site between two α-helices and in front of a β-sheet. In the binding site, the substrate forms five hydrogen bonds with the enzyme: one from Asp119, two from Asp120, and two from Asn33. Two water molecules, 2024 and 2134, also form hydrogen bonds with the substrate

3ZR0 consists of two entire systems, labeled chains A and B, with each system containing one molecule of MTH1 plus the substrate 8-oxo-dGMP as well as sulfate ions and a large number of water molecules. The two systems were separated, each system was then hydrogenated, and various sites were ionized, mainly by the formation of salt bridges. Within the binding site (see Fig. 1), the distance between O_δ_ on Asp119 and O_6_ in 8-oxo-dGMP, labeled 8OG-1157, was unusually small, indicative of the presence of an anion. The likelihood that residue Asp119 was protonated [25] or deprotonated [24] was examined, and calculations [6] predicted that the proton between the two oxygen atoms was nearer to the oxygen of the carboxylate group, so the anionic charge was assigned to O_6_ of 8OG-1157 by deleting the hydroxyl hydrogen. Other sites that might be ionized in vivo were identified, but, because the effect on the binding site of these potentially ionized moieties was expected to be very small, no attempt was made to determine whether or not they should be ionized. The final result of the various ionizations was that each system had a net charge of −1, and the empirical formulae were C_817_H_1514_N_214_O_382_S_8_P for system A and C_798_H_1352_N_206_O_314_S_9_P for system B.

### Conventional geometry optimization

The conventional method for generating a starting model for use in simulations uses an unconstrained global optimization. This was performed on system A. Unfortunately, even though a fully optimized stationary point on the potential energy surface was achieved, the root-mean-square deviation (RMSD) between the PM7 and X-ray geometries of the substrate plus binding site was 0.797 Å. This difference was so great that errors due to wrongly positioned residues in the active site would likely render any further work invalid, and consequently this approach was abandoned.

### Modified geometry optimization

To a large degree, much of the geometric difference in the binding site could be attributed to the consequences of the motion of residues that were not involved in binding; in general [26], this motion is both large and involves very little energy. If this motion could be reduced without compromising the integrity of the model of the binding site, then the usefulness of the model would be increased. To explore this possibility, the 8-oxo-dGMP substrate was replaced by dGMP, the geometry reoptimized, and the resulting fully optimized geometries of 8-oxo-dGMP and GMP compared. After deleting the substrates and the residues that composed the binding site, the RMSD between the two systems was 0.002 Å. That is, the effect of replacing the substrate 8-oxo-dGMP by dGMP was to cause atoms that were not in the binding site to move by an average of only about 0.002 Å, a completely insignificant amount.

Having established that changes in the binding site would have a negligible effect outside the binding site, the task of reducing the RMSD error in the binding site was then addressed.

A recent technique [27] for refining protein crystal structures involves applying a weak restraining force to the optimization process. The effect of this force is to apply an energy penalty which would increase as the difference between the calculated and reference geometries, here the hydrogenated PDB geometry, increases. This technique has an important advantage in that the large distortions in overall protein geometries resulting from the use of semiempirical methods can easily be eliminated with only a minimal energy penalty. Using this technique, an attempt was made to improve the accuracy of prediction of the molecular structure within the binding site.

A restraining force of 3 kcal mol^−1^ Å^−1^ was applied and the geometries of systems A and B were reoptimized. In order to avoid the atoms in the binding site being influenced by the restraining force, a second geometry optimization that did not use the restraining force, and involved only those atoms that were within 5.0 Å of any atom in the substrate, was then carried out. During this process the positions of all other atoms were kept fixed. Following this operation, the RMSD for the binding site of system A decreased from 0.797 Å to 0.319 Å. The resulting structures were ideally suited for use as models of the binding site, in that the geometry of the binding site was in good agreement with the X-ray structure, and therefore more realistic, and all the atoms within the binding site were unconstrained, so that simple geometric operations—in particular mutations—could be performed.

**Fig. 2:**
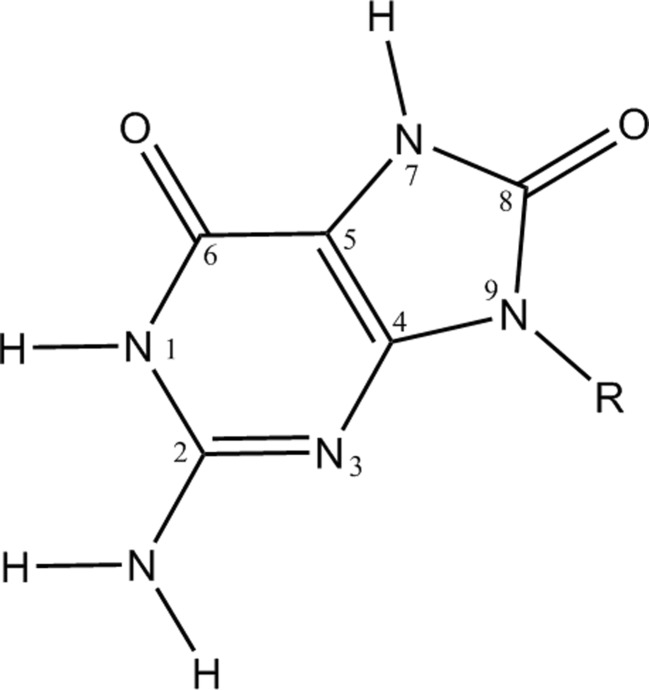
Atom numbering system for 8-oxo-guanine

Four other systems were prepared from these highly optimized structures. Two of these were formed when 8-oxo-dGMP was mutated to dGMP by deleting H_7_ and converting O_8_ to H_8_ (see Fig. 2 for atom numbering), followed by the exhaustive optimization of the positions of all atoms in the binding site. The other two were formed by deleting the 8-oxo-dGMP: this operation resulted in the formation of a system with a net charge of zero, but with both Lys23 and Asp119 ionized. Exhaustive geometry optimization was then performed on all atoms within 5.0 Å of where the 8-oxo-dGMP had been.

For convenience, these six systems are labeled A-8OG, A-GMP, A-NUL, B-8OG, B-GMP, and B-NUL. When reference is made to both systems, the labels 8OG, GMP, and NUL will be used.

### Geometry optimization of the binding site

One approach to increasing the precision would be to restrict geometry optimization operations to only those atoms that were involved in the binding site. This avoids two sources of imprecision that occur when global optimizations are used. First, when global geometry optimizations are performed on protein systems, the calculated Δ*H*_f_ fluctuates from cycle to cycle. Fluctuations of this type are a result of the use of finite criteria for the various steps involved in calculating the geometry changes, and have a magnitude comparable to those of the noncovalent interactions of interest. Second, the possibility exists that, as a consequence of a minor modification being made to a geometry and a global optimization then being re-run, the new geometry might be several kcal mol^−1^ more stable than expected. This could occur when, for example, a new hydrogen bond forms as a result of a geometry reoptimization. Such a bond might be completely unrelated to the modification made, but its formation would be sufficient to render any resulting energies useless.

To verify that geometry optimizations using only the atoms in the binding site would result in an increase in precision, ten of the residues that were outside the 5.0 Å limit were mutated to an alanine by replacing the side chain with a methyl group. Having established that changing the substrate had little effect on the positions of atoms that were far from the binding site, the effect on the binding site of modifying (i.e., mutating) residues that were far from the binding site would also be expected to be very small. The residues chosen were Val96, Ser98, Asp99, Glu100, Met101, Cys104, Trp105, Phe106, Gln107, Leu108, and Gln110. One at a time, each of these residues was mutated and a limited geometry optimization involving only the mutated residue and the atoms in the binding site was performed. Using this set, the largest change in the difference in the heats of formation of the two systems B-8OG and B-GMP was 0.13 kcal mol^−1^, with the average unsigned change being 0.05 kcal mol^−1^. Based on this, the conclusion was made that a restricted optimization would result in an improvement in precision from about 2 kcal mol^−1^ to less than 0.2 kcal mol^−1^.

### Increasing precision by using “exact” fragments

Subsequent work indicated that even this improved precision might not be sufficient. If the assumption was made that the binding sites in systems A and B were identical, then the various energy differences calculated for A and B should also be identical. This was not observed. Instead, the results of pilot mutation experiments showed that there were significant differences between the two systems.

To eliminate as much of the remaining imprecision in the calculated energies as possible, a new protocol was developed that was designed to improve the precision still further. This involved the following three conditions:

- For each system being modeled, the geometry was based on one of the six starting geometries: A-8OG, A-GMP, A-NUL, B-8OG, B-GMP, and B-NUL.
- Each modification involved the mutation of a residue. Within the set A-8OG, A-GMP, and A-NUL, regardless of which system was being modeled, the geometry of the mutated residue was exactly the same. The same constraint was used for all B systems.
- None of the atoms were allowed to move. That is, only single-point calculations were run.

It was assumed that these conditions did not introduce any significant energy terms because every mutation resulted in the elimination of the corresponding residue–substrate interaction. To verify that the use of a single mutated residue—regardless of whether it originated from a 8OG or a GMP complex—was justified, tests were performed in which a mutated residue from one complex (such as A-8OG) was placed in the other complex (for example A-GMP), and vice versa, and the resulting energies compared. All differences were less than 0.1 kcal mol^−1^, thus validating the assumption and also confirming that the use of the new protocol caused the errors in precision to be reduced by 50 %.

Having established that a constrained optimization resulted in a useful precision for both geometry and Δ*H*_f_ calculations, and that no artefacts had been introduced, the only conclusion that could be made regarding the differences in the binding energies in systems A and B was that they were not an artefact of the calculation—they were being caused by differences in the two systems.

## Results

### Substrate on its own

Prior to a substrate binding to the enzyme, it would likely be in solution in the cytoplasm or in the nucleus (i.e., be in aqueous media), and would thus also exist as the anion. In solution, both substrates could exhibit keto–enol tautomerization, and, because of flexibility around the deoxyribose–guanine bond, would also exhibit *syn*-*anti* conformational flexibility. This would give rise to a large number of stable minima, of which the most important eight for 8-oxo-dGMP and the most important four for dGMP are shown in Table 1. PM7 predicts that the most stable structure for 8-oxo-dGMP, the *syn*-keto-keto, would be 2.35 kcal mol^−1^ more stable than the *anti*-keto-keto, and that the most stable structure for dGMP would also be the *syn*-keto, with the *anti*-keto being 0.32 kcal mol^−1^ higher in energy. A similar prediction was obtained using the B3LYP [28] functional with the DGDZVP basis set in Gaussian 09 [29] for both 8-oxo-dGMP and dGMP, with the *syn*-keto-keto being 3.39 kcal mol^−1^ more stable than the *anti*-keto-keto and the *syn*-keto being more stable by 1.74 kcal mol^−1^ than the *anti*-keto, respectively.

**Table 1:** PM7 heats of formation of substrate anions in solution

PO_4_	6	8	PM7 Δ*H*_f_	Diff.	B3LYP total energy^†^	Diff.
8-Oxo-dGMP						
*syn*	keto	keto	−517.99	0.00	−1606.299260	0.00
*anti*	keto	keto	−515.65	2.35	−1606.293859	3.39
*syn*	enol	keto	−509.38	8.62	−1606.288475	6.77
*anti*	enol	keto	−506.35	11.64	−1606.283450	9.92
*syn*	keto	enol	−500.09	17.91	−1606.264663	21.71
*anti*	keto	enol	−508.91	9.08	−1606.272482	16.80
*syn*	enol	enol	−490.55	27.44	−1606.255105	27.71
*anti*	enol	enol	−509.05	8.94	−1606.279088	12.66
dGMP						
*syn*	keto		−459.71	0.00	−1531.035079	0.00
*anti*	keto		−459.39	0.32	−1531.032314	1.74
*syn*	enol		−449.84	9.87	−1531.022910	7.64
*anti*	enol		−449.42	10.29	−1531.020577	9.10

The *PO*_*4*_ orientation is relative to the guanine. *6* and *8* refer to the atom numbers of the possible tautomers. *Diff.* is the Δ*H*_f_ relative to the lowest-energy structure. All energies are in kcal mol^−1^, except for the B3LYP total energies, which are in au.

^†^ Obtained using the DGDZVP basis set.

Both PM7 and B3LYP predict that, in solution, the most stable conformer of 8-oxo-dGMP and dGMP would be *syn*, but all the energy differences between the *syn* and *anti* conformations of the keto tautomers were so small that little significance could be attached to the prediction of the most stable conformer. Other factors could change the relative energies, of which the most important are the limited accuracy of the methods used and the possibility of other environmental effects, such as solvated counterions near to the substrate. Either of these factors could be responsible for changes on the order of a few kcal mol^−1^ in the relative energies of the conformers.

Both PM7 and B3LYP also predict that, in solution, the most stable tautomers of 8-oxo-dGMP and dGMP would be keto. In the B3LYP calculation, the energy difference between the lowest-energy tautomer and the most stable enol tautomer of 8-oxo-dGMP was 6.77 kcal mol^−1^. This difference was so large that the previously described factors that influence energy differences would be unlikely to reverse the order of tautomers. This result is corroborated by a report of a high-level calculation [30] that “(the) enol tautomer … is not stable in the aqueous phase. It is 8.7 kcal mol^−1^ higher in free energy than (the keto form) leading to a population in the aqueous phase of 4 · 10^−7^.”

Although the most stable solution-phase geometry of 8-oxo-dGMP was predicted to be *syn*, the structure of 8-oxo-dGMP found in 3ZR0 was in the *anti* conformation. Presumably, the observed conformation of the oxidized substrate would be the result of features within the binding-site environment that gave it extra stability.

Given that the *syn* conformation of dGMP in the aqueous phase was only 0.32 kcal mol^−1^ less stable than the *anti*, and assuming that both 8-oxo-dGMP and dGMP would be stabilized in the same way in the binding-site environment, it follows that dGMP would also exist in the binding site in the *anti* conformation. Because of this, no further consideration was given to the *syn* conformers, and all further reference to either substrate in the binding site should be regarded as referring to the *anti* conformer.

At physiological pH, both substrates would most likely exist as the monoanion, with the negative charge being on the phosphate group, –[HPO_4_]^−^, and the guanine group at the other end of the substrate being uncharged. Based on the structure of the complex in 3ZR0, a negative charge must exist in the assembly composed of Asp119, Asp120, and the guanine of the substrate. A precise definition of the location of this charge at one or the other of the aspartic acid residues or at the guanine group could not be made [26] because of the very strong hydrogen bonding that was present; however, once the substrate was separated from the binding site so that the guanine became neutral, the anionic site would necessarily become localized on the two Asp residues in order for the charge to be conserved.

### MTH1 on its own

**Fig. 3:**
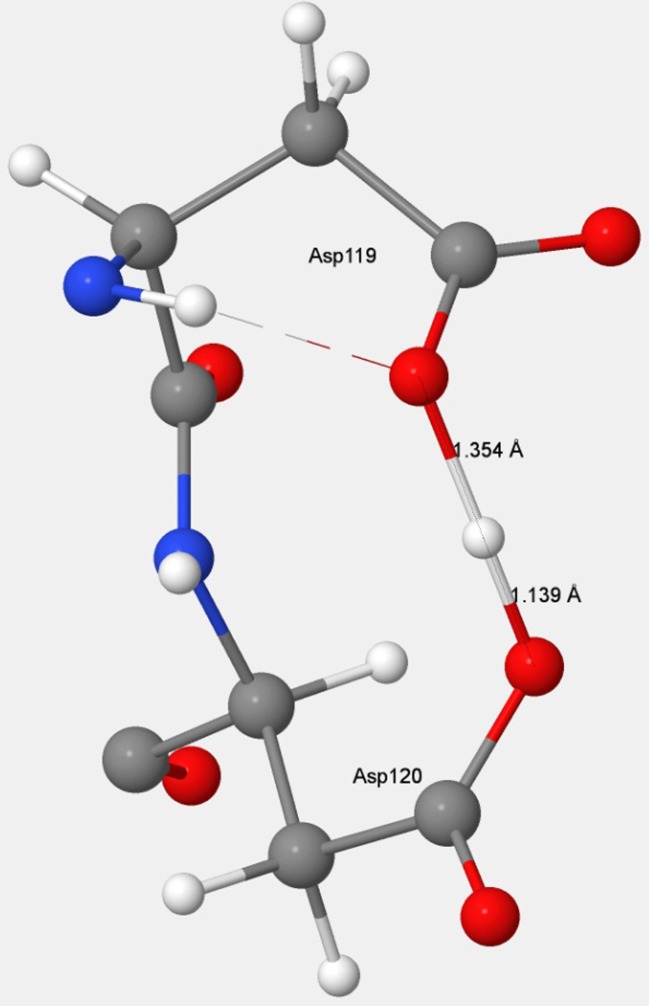
The D119–D120 anion in MTH1. The position of the ionizable hydrogen atom suggests that Asp119 exists as the carboxylate anion and that Asp120 exists as the neutral carboxylic acid

An important geometric change occurred in MTH1 when the substrate was removed from the binding site. Unless another anion migrated in to replace the departing guanine anion, its departure would result in the unit negative charge becoming localized on the two Asp residues. This would give rise to the structure shown in Fig. 3. In PDB entry 3ZR1, an MTH1 structure where the normal substrate is missing, an acetic acid molecule located near to the Asp–Asp pharmacophore suggests the presence of a negative charge in that vicinity, so the inference could be made that a unit negative charge would also exist in the vicinity of Asp119–Asp120 in the current system.

In addition, the departure of the phosphate on the substrate, which had formed a salt bridge with the ionized site in Lys23, resulted in significant motion of the water molecules in the region of N_ζ_ on Lys23. These molecules were near to Glu52 and Glu56, two residues within the catalytic Nudix box in MTH1, but, as most of the atoms in these residues were outside the 5.0 Å limit, the geometries of these residues were not affected significantly as a result of the departure of the substrate.

### Docking of substrate

In 3ZR0, 8-oxo-dGMP is docked in the binding site. This provided an opportunity to compare the observed and predicted structures of the interface between the binding site and the substrate. With one exception, all the interactions had the expected geometry.

In system B, PM7 predicted the N_δ2_–N_3_ hydrogen-bond distance between Asn33 and 8OG-1157 to be 0.4 Å too large, although the other hydrogen bond, between O_δ1_ and N_2_, was similar in length to that in the X-ray structure. In addition, PM7 predicted the formation of a normal hydrogen bond between N_δ2_ and O_4′_, the oxygen atom in the deoxyribose ring. There was no indication of the presence of such a hydrogen bond in the X-ray structure.

Analysis of the environment of Asn33 revealed the presence of a water molecule in A-8OG (H_2_O-2024) for which no equivalent was present in B-8OG. In A-8OG, this water molecule formed two hydrogen bonds, one with Asn33 N_δ2_ and one with 8OG-1157 O_4′_, leading to the conclusion that the incorrect structure predicted for B-8OG was a result of the absence of that water molecule from its X-ray structure.

#### Stabilization due to the binding pocket

For the purposes of this study, the stabilization energy for the substrate 8-oxo-dGMP docked in the binding site of MTH1 was defined as the energy difference between the heat of formation of the separated, solvated components (solvated 8-oxo-dGMP and solvated MTH1) and the heat of formation of the solvated complex. This definition does not include any other species, such as counterions, that might be present; such species would not alter the individual binding energies but would alter the heat of reaction. An estimate of the heat of reaction, Δ*H*_r_, for the formation of the solvated complex was obtained from the heats of formation of A-8OG (−24446.41 kcal mol^−1^), A-NUL (−23840.25 kcal mol^−1^), and 8-oxo-dGMP (−517.99 kcal mol^−1^) via

$$ \varDelta {H}_{\mathrm{r}} = \varDelta {H}_{\mathrm{f}}\left(\mathrm{A}-8\mathrm{O}\mathrm{G}\right)-\left(\varDelta {H}_{\mathrm{f}}\left(8-\mathrm{o}\mathrm{x}\mathrm{o}-{\mathrm{dGMP}}_{\mathrm{Aq}}\right) + \varDelta {H}_{\mathrm{f}}\left(\mathrm{A}-\mathrm{N}\mathrm{U}\mathrm{L}\right)\right) = -88.17\ \mathrm{kcal}\ {\mathrm{mol}}^{-1}. $$

**Fig. 4:**
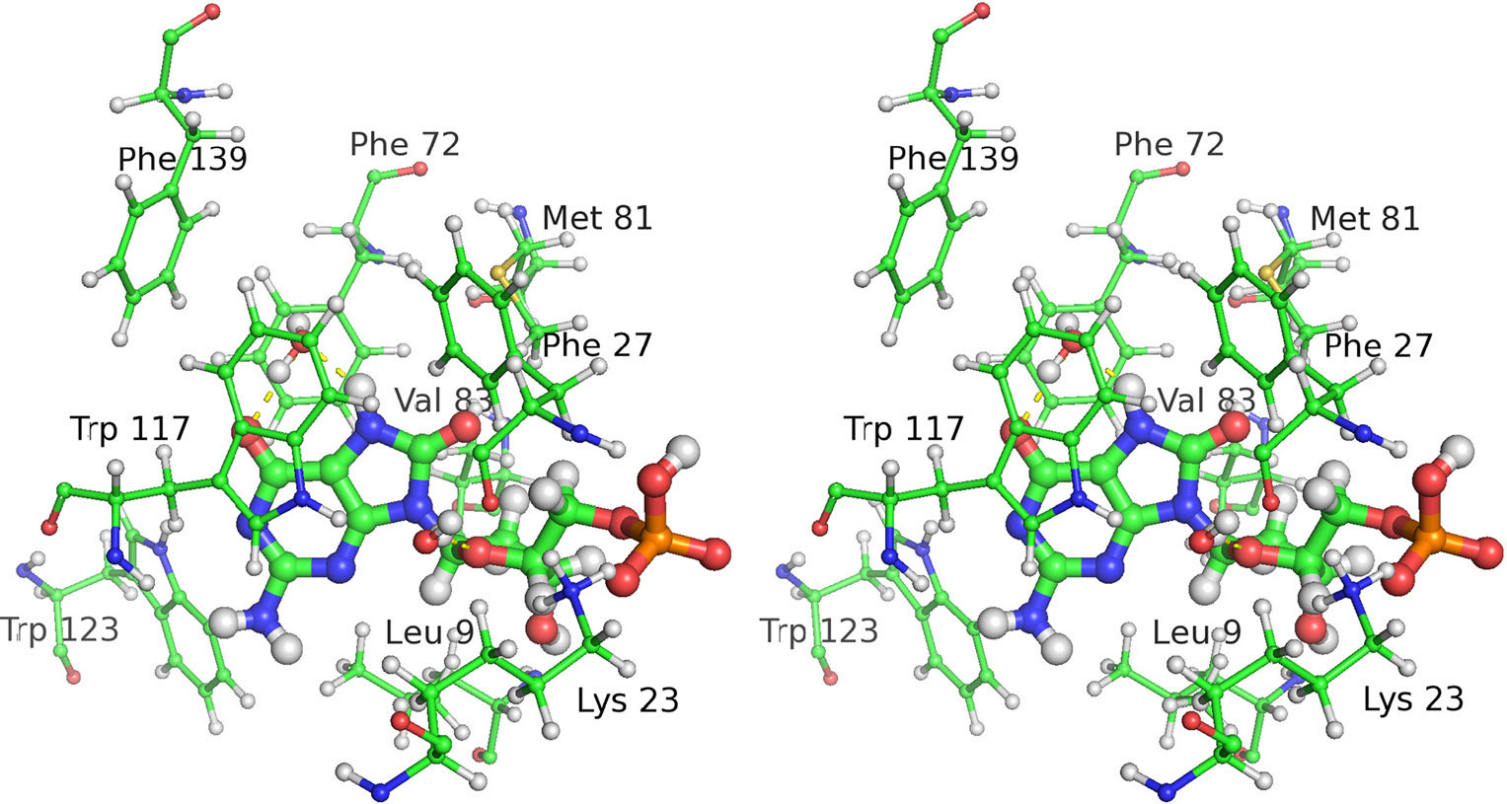
Stereo view of residues in the binding pocket that do not form hydrogen bonds with the 8-oxo-dGMP substrate

An alternative method of calculating the heat of reaction would be to evaluate the sum of the energy terms for the various residue–substrate interactions in the binding pocket. In MTH1, this pocket is composed of 11 residues, which can be divided into two groups: a set of three hydrogen-bonding residues: Asp119, Asp120, and Asn33; and a set of eight π-stacking and other hydrophobic residues: Leu9, Phe27, Phe72, Met81, Val83, Trp117, Trp123, and Phe139 (see Fig. 4). Although not part of the binding pocket, a twelfth residue, Lys23, does form a strong salt bridge with the phosphate group, and was included in this study for completeness. In A-8OG there was one water molecule, H_2_O-2134, that would be involved in hydrogen bonding to the substrate; the equivalent molecule was not resolved in B-8OG, so this molecule was added to B-8OG for consistency. This molecule is important in that its presence would stabilize both the oxidized [24] and the native substrate: in its absence the oxidized substrate atoms H_7_ and O_6_ atoms and the native substrate atoms O_6_ and N_7_ would be in strongly hydrophobic (i.e., unrealistic) environments. Although somewhat different in principle from the other mutations where changes were made to the side chains of residues, the presence of this water molecule introduced no new issues that might compromise the significance of any resulting energies or geometries, so energy contributions due to the interaction of H_2_O-2134 with the substrates were evaluated in a similar way to those of the residues.

All the residues in the binding pocket interact via their side chains. If these were replaced by a much smaller side chain so that a gap or space was introduced between the residue and the substrate, then the corresponding noncovalent interactions would become insignificant. This operation would be the in silico analog to the experimental process of mutation analysis when investigating the role of individual residues, the main difference being that obtaining results using experimental methods is both more difficult and time-consuming. Using system A, two starting points were used for this comparison, one being the isolated MTH1 protein and the other the MTH1 protein with 8-oxo-dGMP docked in the binding site. Each of the 12 residues was mutated one at a time to replace the side chain with a smaller group. With the exception of Lys23, which exists as the cation as one-half of a salt bridge, the replacement was a methyl group; Lys23 was mutated by replacing the terminal –NH_3_^+^ group with a hydrogen atom, this being the smallest change that would achieve the objective of eliminating the interaction between the side chain and substrate.

An estimate of the binding energy *B*_R_ attributable to a residue R could then be obtained from the difference in the resulting heats of formation, as shown in Eq. 1, where Δ*H*_f_(MTH1 + substrate) and Δ*H*_f_(NUL) are the heats of formation of the unmutated systems and Δ*H*_f_(MTH1 + substrate)_R_ and Δ*H*_f_(NUL)_R_ are the heats of formation of the complex in which residue R was mutated.

1 $$ {B}_{\mathrm{R}}=\left(\varDelta {H}_{\mathrm{f}}\left(\mathrm{M}\mathrm{T}\mathrm{H}1+\mathrm{substrate}\right) - \varDelta {H}_{\mathrm{f}}\left(\mathrm{N}\mathrm{U}\mathrm{L}\right)\right) - \left(\varDelta {H}_{\mathrm{f}}{\left(\mathrm{M}\mathrm{T}\mathrm{H}1+\mathrm{substrate}\right)}_{\mathrm{R}}-\varDelta {\mathrm{H}}_{\mathrm{f}}{\left(\mathrm{N}\mathrm{U}\mathrm{L}\right)}_{\mathrm{R}}\right) $$

Or, after substituting for the heats of formation of the unmutated systems,

$$ {B}_{\mathrm{R}}=\varDelta {H}_{\mathrm{f}}{\left(\mathrm{N}\mathrm{U}\mathrm{L}\right)}_{\mathrm{R}}-\varDelta {H}_{\mathrm{f}}{\left(8\mathrm{O}\mathrm{G}\right)}_{\mathrm{R}} - 606.16, $$

and, for the A-GMP system, where Δ*H*_f_(A-GMP) = −24380.00 kcal mol^−1^,

$$ {B}_{\mathrm{R}}=\varDelta {H}_{\mathrm{f}}{\left(\mathrm{N}\mathrm{U}\mathrm{L}\right)}_{\mathrm{R}}-\varDelta {H}_{\mathrm{f}}{\left(\mathrm{G}\mathrm{M}\mathrm{P}\right)}_{\mathrm{R}}-539.75. $$

All individual energy contributions are shown in Table 2. If the assumption were to be made that the interactions between the substrate and the individual parts of the binding pocket were independent, then the sum of the contributions for A-8OG would add up to −70.59 kcal mol^−1^. This is smaller by 17.58 kcal mol^−1^ than the heat of reaction obtained earlier (−88.17 kcal mol^−1^). In part, this difference could be attributed to the extra stabilization resulting from the transfer of the proton from O_6_ on 8-oxo-dGMP to Asp119 that takes place in the docked complex, as this energy term would not be reproduced by the single-residue mutations.

**Table 2:** Energy contributions to the stabilization of 8-oxo-dGMP and dGMP, in kcal mol^−1^

Residue	Δ*H*_f_(A-NUL)_R_	Δ*H*_f_(A-8OG)_R_	Δ*H*_f_(A-GMP)_R_	Stabilization energy	
				A-8OG^a^	A-GMP^b^
Asp119	−23644.90	−24328.99	−24262.90	−6.07^c^	−5.75^d^
Asp120	−23655.84	−24330.10	−24263.40	−15.89^c^	−16.18^d^
Asn33	−23773.56	−24365.65	−24300.19	−14.07	−13.12
Leu9	−23814.97	−24415.26	−24349.13	−5.87	−5.59
Lys23	−23893.82	−24492.09	−24426.03	−7.89	−7.55
Met81	−23828.01	−24434.66	−24369.51	+0.49	+1.76
Trp117	−23847.76	−24449.51	−24385.23	−4.41	−2.28
Phe27	−23854.25	−24457.49	−24392.04	−2.92	−1.97
Val83	−23821.84	−24425.62	−24359.08	−2.38	−2.51
Phe72	−23847.94	−24449.37	−24385.15	−4.73	−2.54
H_2_O-2134	−23765.50	−24366.34	−24301.07	−5.31	−4.17
Trp123	−23845.34	−24450.28	−24382.71	−1.21	−2.37
Phe139	−23842.76	−24448.58	−24383.16	−0.33	+0.66

^a^ Energy = Δ*H*_f_(A-NUL)_R_ − Δ*H*_f_(A-8OG)_R_ − 606.16.

^b^ Energy = Δ*H*_f_(A-NUL)_R_ − Δ*H*_f_(A-GMP)_R_ − 539.75.

^c^ Energy = Δ*H*_f_(A-NUL)_R_ − Δ*H*_f_(A-8OG)_R_ − 690.15. See text for details.

^d^ Energy = Δ*H*_f_(A-NUL)_R_ − Δ*H*_f_(A-GMP)_R_ − 623.74. See text for details.

Note: Δ*H*_f_ of the unmodified systems were Δ*H*_f_(A-NUL) = −23840.25, Δ*H*_f_(A-8OG) = −24446.41, and Δ*H*_f_(A-GMP) = −24380.00 kcal mol^−1^; for Asp 119 and Asp 120, Δ*H*_f_(A-NUL) = −23756.26 kcal mol^−1^.

### Roles of Asp119 and Asp120

**Fig. 5:**
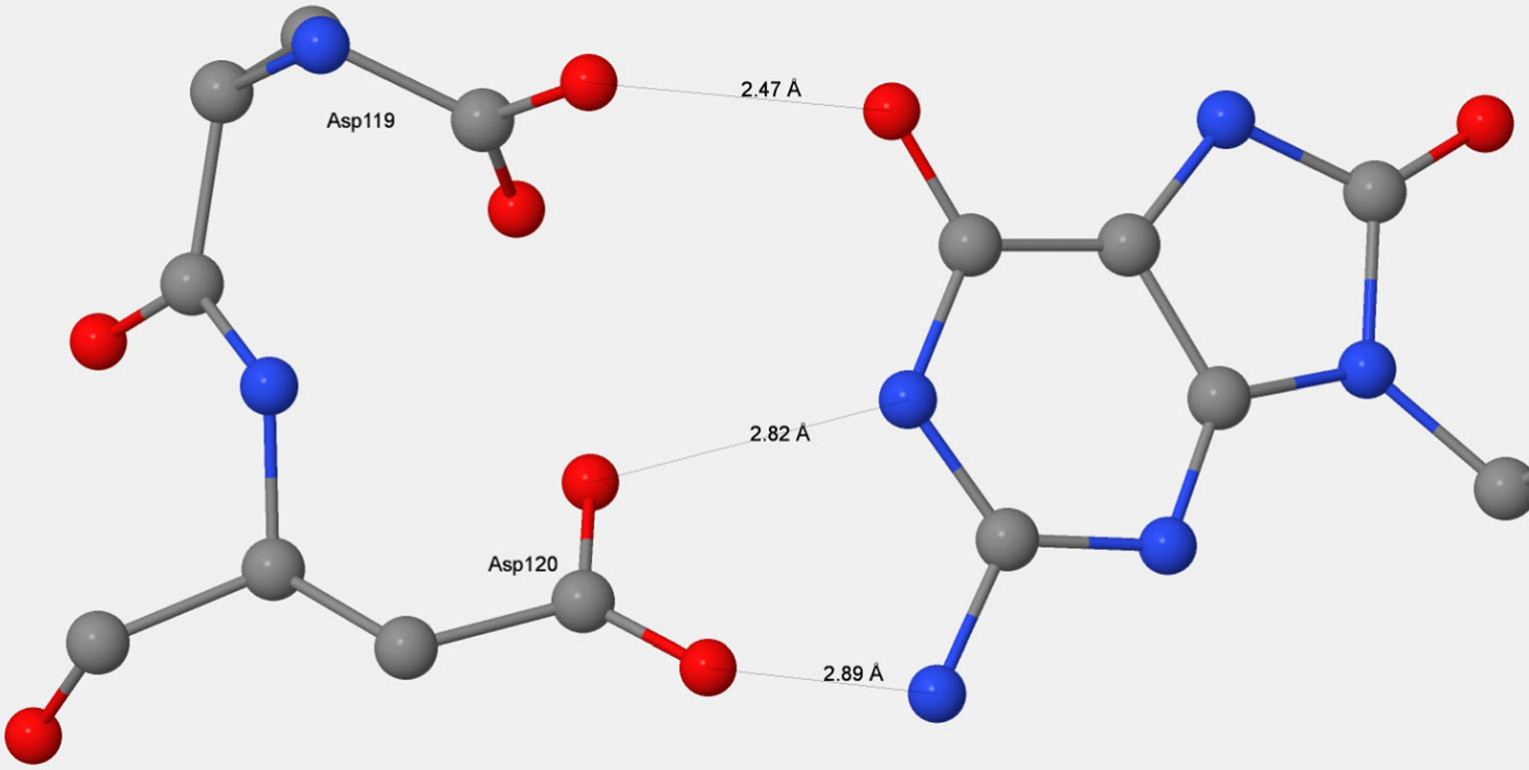
Hydrogen-bonding structure in the D119–D120–guanine complex

Residues that form the recognition pocket could only bond with the substrate through noncovalent interactions; of these, hydrogen bonds would be the strongest, so it might be expected that Asp119, Asp120, and Arg33, contributing a total of five hydrogen bonds, would be the most stabilizing. This was true for Arg33, which formed two strong hydrogen bonds that stabilized A-8OG by 14.07 kcal mol^−1^, but when Asp119 and Asp120 were mutated using the same procedure as employed for all the other residues, the results obtained did not indicate the presence of strong hydrogen bonds. For Asp120, even though two strong hydrogen bonds were formed (see Fig. 5), the stabilization energy was only −8.52 kcal mol^−1^. For Asp119, which contributes the shortest—and therefore presumably the strongest—hydrogen bond, not only was there no stabilization, but the presence of that hydrogen bond resulted in a destabilization of 5.02 kcal mol^−1^.

This unexpected result warranted a re-examination of the Asp–Asp pharmacophore, and led to a completely different interpretation of the interaction with the guanine.

When the substrate was not docked in the binding site, the Asp–Asp pharmacophore would presumably have a net unit negative charge, and the remaining ionizable hydrogen atom would be located somewhere between the two carboxylate groups, as shown in Fig. 3. Its position had been predicted [26] to be much nearer to an oxygen on Asp120 than to that on Asp119, which would imply that Asp120 should be regarded as a neutral carboxylic acid, and that Asp119 contained an anionic carboxylate group, –COO^−^.

In all other mutations, the Asp–Asp anion pharmacophore would remain unaffected, but in the two mutations that involved either Asp119 or Asp120, this structure would be destroyed.

**Fig. 6:**
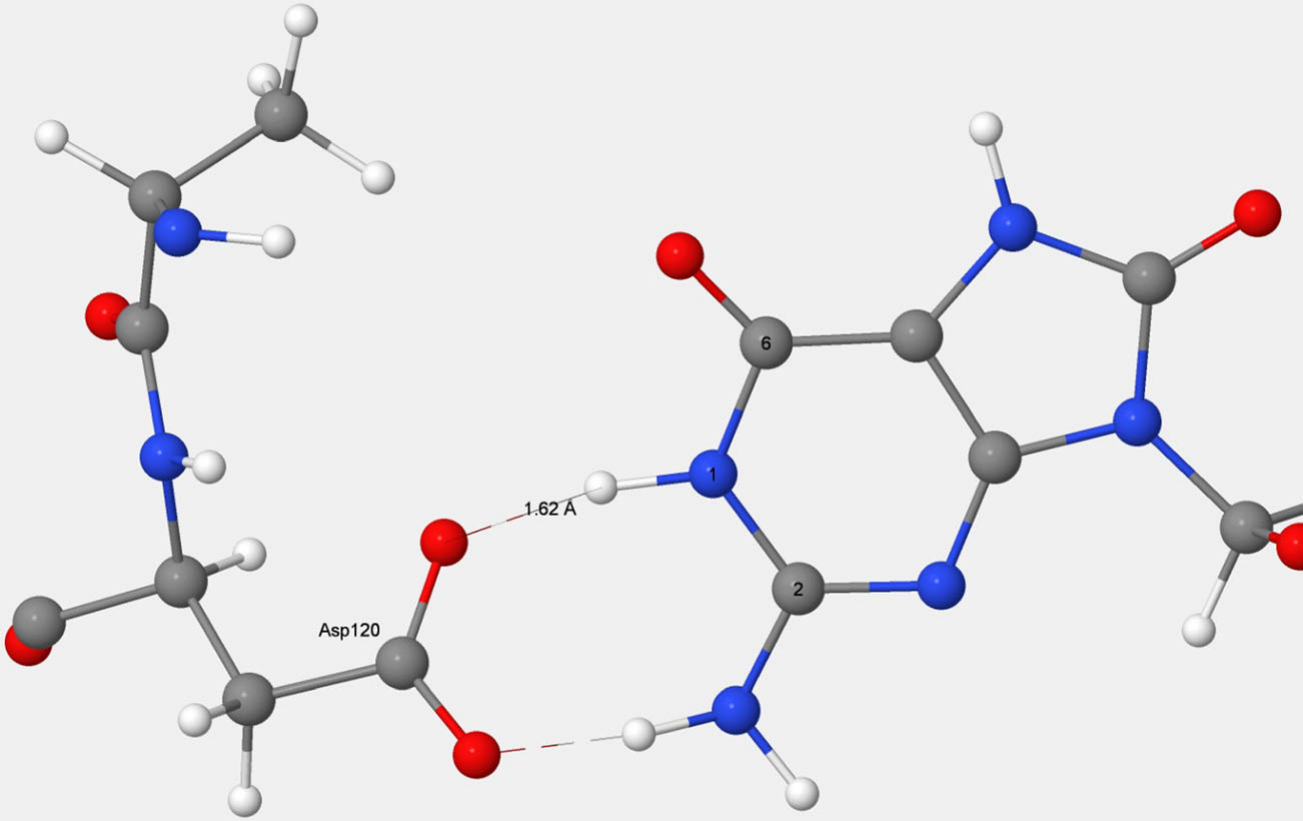
Mutation D119A in MTH1 + 8-oxo-dGMP. In the D119A mutation, Asp120 spontaneously ionizes to form the carboxylate, which hydrogen bonds to neutral guanine

**Fig. 7:**
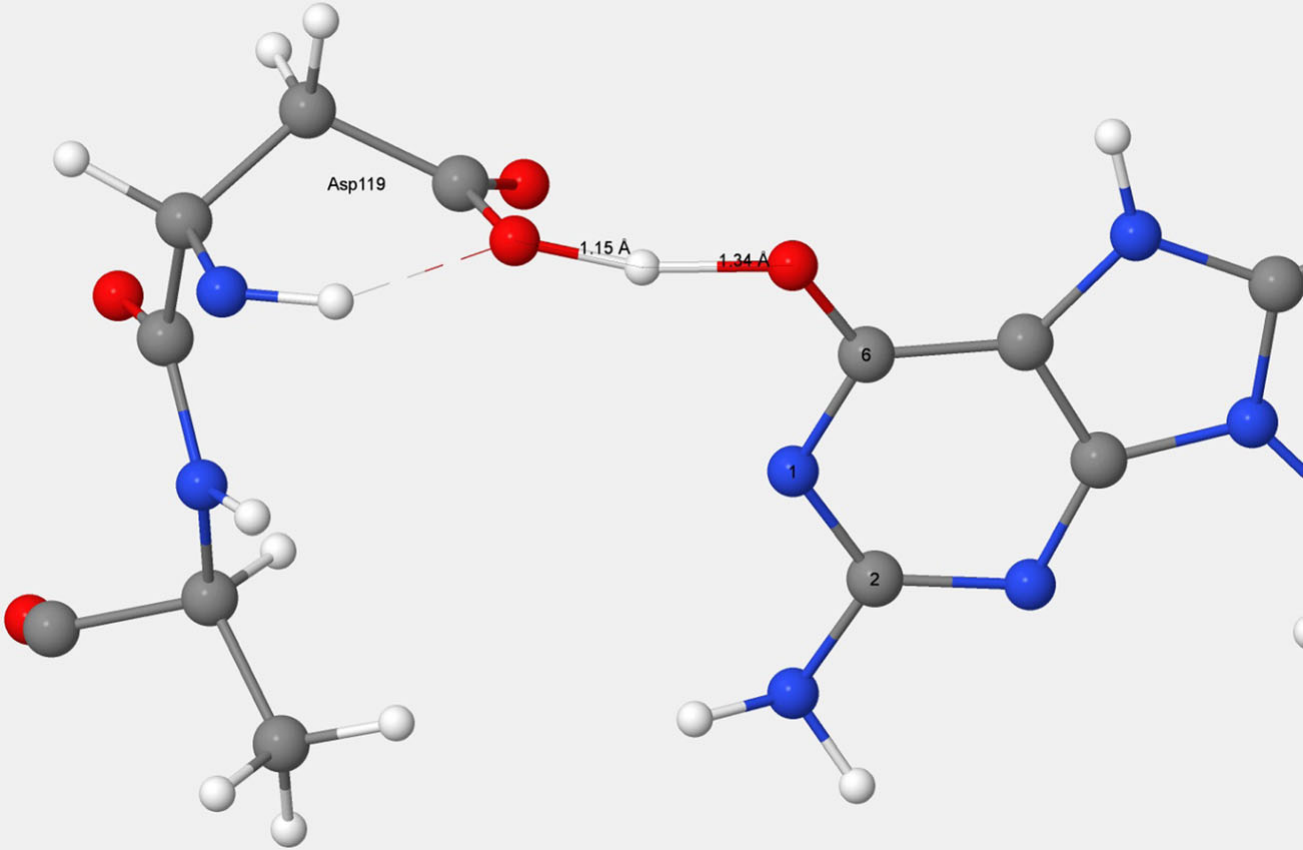
Mutation D120A in MTH1 + 8-oxo-dGMP. In the D120A mutation, the position of the ionizable hydrogen atom suggests that Asp119 remains a neutral carboxylic acid which forms a strong hydrogen bond with the guanine anion

When the D119A mutation was performed on 8OG, the ionizable hydrogen on Asp120 migrated to N_1_, resulting in the Asp120 becoming an anion and the guanine becoming neutral, as shown in Fig. 6. In natural MTH1, strong hydrogen bonds exist between the anionic guanine and both Asp119 and Asp120. When the stabilization due to the presence of the Asp119 carboxylic acid side chain was removed in the D119A mutation, the equilibrium shifted so that D120 became anionic and the guanine became neutral. This behavior could be contrasted with the D120A mutation, where Asp119 was essentially unaffected; it remained as the neutral carboxylic acid hydrogen bonding to the anionic guanine, as shown in Fig. 7.

In both mutations, the Asp–Asp anionic pharmacophore was replaced by a structure in which one (in the case of D120A) or two (in the case of D119A) strong hydrogen bonds were formed with the guanine.

An estimate of the energy difference between the bonding of Asp119 to guanine and the bonding of Asp120 to guanine was obtained by calculating the interaction of acetic acid with a guanine molecule in which a hydrogen bond was formed in the style of Asp119 (that is, to the O_6_ of guanine) and, in a separate calculation, two hydrogen bonds were formed in the style of Asp120. Using PM7, the energy of the Asp120-style system was 9.1 kcal mol^−1^ more stable than that of the Asp119-style system. Using B3LYP and the 6-311G basis set, a qualitatively similar result was obtained, the energy difference being 17.8 kcal mol^−1^. These values were similar to the difference, 13.54 kcal mol^−1^, between the stabilization energies calculated for Asp119 and Asp 120. Both PM7 and B3LYP predicted that, in the Asp119 form, the proton would be nearer to the acetate group, and in the Asp120 form, it would be nearer to the guanine group. A test was done to confirm that the ionizable hydrogen atoms in the various systems were correctly positioned. Regardless of the initial placement of the ionizable hydrogen atom, optimization of the D119A system always resulted in the proton that was originally on Asp120 moving to be nearer to the guanine. Optimization of the D120A system, again regardless of the initial placement of the proton, always resulted in it moving to be nearer to Asp119 in the mutant.

That both the large difference in stabilization energy and the position of the ionizable hydrogen atom could be reproduced in a simple system using PM7 and B3LYP supports the prediction of the energies and structures in the Asp119–Asp120–guanine system.

Together, these results allow an explanation to be given for the observed decrease in stabilization resulting from the D119A mutation.

When 8-oxo-dGMP or dGMP binds to MTH1, a proton on guanine migrates to the Asp119–Asp120 carboxylate–carboxylic acid complex (Fig. 3), effectively destroying the hydrogen bond that was present and replacing it with three new hydrogen bonds connecting the guanine and the now-separated Asp119 and Asp120 (Fig. 5). This process would result in a net decrease in energy, with the increase in energy due to the destruction of the carboxylate–carboxylic acid hydrogen bond being more than offset by the decrease in energy resulting from the formation of three hydrogen bonds. In the D120A mutation, the docked system would have only one hydrogen bond, from Asp119 to the guanine. In the unmutated docked system, there would be three hydrogen bonds, so the presence of Asp120 would be responsible for a net increase of two hydrogen bonds. In D119A, the docked system has two hydrogen bonds, both from Asp120, so the presence of Asp119 resulted in an increase of only one hydrogen bond. Mutation D119A resulted in a destabilization of 5.02 kcal mol^−1^ because the single hydrogen bond that was formed with the substrate would not be as strong as the hydrogen bond to Asp120 that was destroyed.

An estimate was made of the stabilization energy that Asp119 and Asp120 would provide if the extra stabilization caused by the existence of the carboxylate–carboxylic acid interaction in MTH1 was removed. This estimate used Asp99, a surface residue where the carboxylic acid group pointed away from the rest of the enzyme and therefore, being exposed to the dielectric of the implicit solvation, would be representative of an isolated acid group. The mutant D99A had a Δ*H*_f_ that was 10.25 kcal mol^−1^ lower than that of the D120A mutant. Thus, 10.25 kcal mol^−1^ represents the extra stabilization of the unmutated MTH1 due to the presence of the carboxylate–carboxylic acid interaction, and should therefore be subtracted from the stabilization energies of Asp119 and Asp120. After this correction was made, the stabilization energy attributable to Asp119 became −5.23 kcal mol^−1^, and for Asp120, −18.77 kcal mol^−1^.

An alternative to using this ad hoc and somewhat complicated correction for these two residues was to model the various systems as if the proton on Asp119 remained on that residue when the substrate was removed. The separated species would then consist of the NUL system with a unit positive charge due to Lys23, and the substrate with anionic charges on both the phosphate and the guanine groups, for a net charge of −2. This assembly was predicted to have an energy only a few kilocalories above the previous system, and would provide a much simpler proxy for the undocked system. The stabilization energies shown in Table 2 for Asp119 and Asp120 use the results of this option.

Both methods of predicting the stabilization energies of these residues gave similar results, and the large difference predicted in their stabilization energies is supported by the observation of Sakai et al. [31] that the D119A mutation did not alter the activity of 8-oxo-dGTPase, the implication being [25] that D120 was the key element in the binding.

### Roles of the other residues

Asn33 forms two hydrogen bonds with 8-oxo-dGMP: between O_δ1_ and an H_2_ and between H_δ2_ and N_3_. Together, these hydrogen bonds made Asn33 the second most stabilizing of all the residues in the binding site.

Of the remaining residues, Lys23 contributes the most to the stabilization due to salt bridge formation. However, the system being modeled used the assumption that, in the absence of the substrate, the binding pocket would be occupied by water. This assumption implies that no counterion would be present; it is more likely that a salt bridge would form between the Lys23 and an adventitious solute anion, and that Lys23 would be stabilized regardless of whether a substrate is present or not.

Three hydrophobic residues—Leu9, Phe72, and Trp117—are in close proximity to hydrophobic parts of the substrate, and all contributed significantly to the stabilization. All the rest of the residues, except Met81 and Phe139, contributed small but significant amounts to the stabilization. Met81 was unique in that its presence contributed a destabilizing effect on the substrate binding energy. Phe139 also tended to destabilize GMP, and contributed an insignificant amount to the stabilization of 8-oxo-dGMP.

A water molecule, H_2_O-2134, is located in an amphipathic pocket in the binding site, one side of which is a hydrophobic environment composed of residues Phe27, Phe72, and Trp117, while the other side comprises the hydrophilic atoms H_7_ and O_6_ of 8-oxo-dGMP and N_7_ and O_6_ of dGMP. This hydrophilic interaction contributed significantly to the stabilization of both 8-oxo-dGMP and dGMP.

#### Origin of specificity

MTH1 discriminates between the oxidized and normal nucleotide using the binding site, assuming that the more strongly the substrate binds to the enzyme, the more likely it is to be hydrolyzed. An estimate of the total difference in binding energy of the substrates was obtained by comparing the Δ*H*_f_ values of various systems involved in the docking process.

When the substrates were docked in the binding site, the differences in the heats of formation of the systems were Δ*H*_f_(A-8OG) − Δ*H*_f_(A-GMP) = −66.41 kcal mol^−1^ and Δ*H*_f_(B-8OG) − Δ*H*_f_(B-GMP) = −67.26 kcal mol^−1^. Subtracting the difference in energy of the two isolated substrates in water in their lowest-energy configurations, −58.28 kcal mol^−1^, from each of the two previous quantities yields −8.13 kcal mol^−1^ for system A and −8.98 kcal mol^−1^ for system B, which represent the calculated total specificity of MTH1. This is the value that can be attributed to the extra stabilization of the oxidized substrate over the native substrate caused by the presence of the enzyme.

An alternative approach to calculating specificity would be to evaluate the contribution arising from each entity in the binding site. For this, the specificity of each residue in the binding site, *S*_R_, was assigned using the same approach used earlier to assign stabilization, the only difference being that GMP was used instead of NUL, as shown in Eq. 2:

2 $$ {S}_{\mathrm{R}}=\left(\varDelta {H}_{\mathrm{f}}\left(8\mathrm{O}\mathrm{G}\right) - \varDelta {H}_{\mathrm{f}}\left(\mathrm{G}\mathrm{M}\mathrm{P}\right)\right) - \left(\varDelta {H}_{\mathrm{f}}{\left(8\mathrm{O}\mathrm{G}\right)}_{\mathrm{R}}-\varDelta {H}_{\mathrm{f}}{\left(\mathrm{G}\mathrm{M}\mathrm{P}\right)}_{\mathrm{R}}\right) $$

or

$$ {S}_{\mathrm{R}}=\varDelta {H}_{\mathrm{f}}{\left(\mathrm{A}-8\mathrm{O}\mathrm{G}\right)}_{\mathrm{R}}-\varDelta {H}_{\mathrm{f}}{\left(\mathrm{A}-\mathrm{G}\mathrm{M}\mathrm{P}\right)}_{\mathrm{R}} - 66.41 $$

and

$$ {S}_{\mathrm{R}}=\varDelta {H}_{\mathrm{f}}{\left(\mathrm{B}-8\mathrm{O}\mathrm{G}\right)}_{\mathrm{R}}-\varDelta {H}_{\mathrm{f}}{\left(\mathrm{B}-\mathrm{G}\mathrm{M}\mathrm{P}\right)}_{\mathrm{R}} - 67.26. $$

Individual contributions of the residues in the binding site to the specificity are given in Table 3. If the assumption were to be made that the interactions between the substrate and the individual parts of the binding pocket are independent, then the sum of the specificities would add to the total specificity.

**Table 3:** Energy contributions to the specificity of the substrates due to systems A and B, in kcal mol^−1^

Residue	System A			System B		
	Δ*H*_f_(8OG)_R_	Δ*H*_f_(GMP)_R_	Specificity energy^a^	Δ*H*_f_(8OG)_R_	Δ*H*_f_(GMP)_R_	Specificity energy^b^
Asp119	−24328.99	−24262.90	−0.32	−19307.03	−19240.04	−0.27
Asp120	−24330.10	−24263.40	+0.29	−19298.65	−19231.54	−0.16
Asn33	−24365.65	−24300.19	−0.96	−19351.29	−19285.08	−1.05
Leu9	−24415.26	−24349.13	−0.28	−19392.44	−19325.05	+0.12
Lys23	−24492.09	−24426.03	−0.34	−19471.20	−19404.28	−0.35
Met81	−24434.66	−24369.51	−1.27	−19408.82	−19342.78	−1.23
Trp117	−24449.51	−24385.23	−2.12	−19436.33	−19371.44	−2.38
Phe27	−24457.49	−24392.04	−0.95	−19434.35	−19366.94	+0.14
Val83	−24425.62	−24359.08	+0.13	−19400.40	−19333.20	−0.06
Phe72	−24449.37	−24385.15	−2.19	−19424.21	−19360.74	−3.79
H_2_O-2134	−24366.34	−24301.07	−1.14	−19342.02	−19275.74	−0.99
Trp123	−24450.28	−24382.71	+1.16	−19424.93	−19357.47	+0.20
Phe139	−24448.58	−24383.16	−0.99	−19424.55	−19358.75	−1.47

^a^ Energy = Δ*H*_f_(8OG)_R_ − Δ*H*_f_(GMP)_R_ − 66.41.

^b^ Energy = Δ*H*_f_(8OG)_R_ − Δ*H*_f_(GMP)_R_ − 67.26.

A negative specificity implies that the residue binds to the 8-oxo-dGMP substrate more strongly than it binds to dGMP.

Δ*H*_f_(A-8OG) = −24446.41, Δ*H*_f_(A-GMP) = −24380.00, Δ*H*_f_(B-8OG) = −19421.98, and Δ*H*_f_(B-GMP) = −19354.72 kcal mol^−1^

For system A, these specificities summed to −8.97 kcal mol^−1^ versus a total specificity of −8.13 kcal mol^−1^, and for B the specificities summed to −11.28 kcal mol^−1^ versus a total of −8.98 kcal mol^−1^. Part of the difference between the summation of component specificities and the total specificity would be caused by the energy required to convert the *syn* conformations of 8-oxo-dGMP and dGMP into the *anti* forms. If, instead of the lowest-energy conformation of the isolated substrate being used, the *anti* form was used, then the total specificities would increase to −10.17 for system A and −11.00 kcal mol^−1^ for system B, in somewhat better agreement with the results of the summations.

#### Individual residues

Four residues—Met81, Trp117, Phe72, and Phe139—were in close proximity to the 7 and 8 positions in the substrate, and were all predicted to contribute significantly to the specificity. The sulfur of Met81 was near enough to the atom attached to C_8_ to be directly affected by its partial charge.

Residues Phe72 and Phe139 were predicted to contribute strongly to the specificity, even though, in the region of the 7 position, the distances between these residues and the substrate were too large for the substrate to have a large direct influence. The conjecture had been made [25] that the hydrogen atom of H_2_O-2134 that was not involved in hydrogen bonding to 8-oxo-dGMP would be free to interact with the π-system of Phe72, Trp117, and Phe139, thus stabilizing them. An examination of the environment between these residues and the substrate revealed that H_2_O-2134 was behaving in the way suggested in the conjecture for Trp117 and Phe72, but not for Phe139.

Residue Phe27 was unusual in that its contribution to specificity was very different in the two systems. No definitive reason could be found for this difference, but an examination of the environment of this residue revealed that there was a water molecule, H_2_O-2024, in system A that was absent in system B. The presence of this molecule in system A allowed two hydrogen bonds to form, one between the carbonyl oxygen atom of the Phe27 and the water molecule, and one between the water molecule and the ring oxygen atom of the substrate. This structure might help define the position of the phenyl ring of Phe27, but the significance of this, if any, was not obvious.

Trp123, Leu9, and Val83 are hydrophobic residues that were predicted to have little effect on specificity. This was unexpected. In the absence of any other overriding considerations, evolutionary pressure to improve the efficiency of MTH1 would be expected to result in them being replaced by other residues that would increase the specificity, or, at least, not reduce it.

Hayakawa et al. [32] reported that MTH1 hydrolyzed 8-oxo-GTP at just 2 % of the rate it hydrolyzed 8-oxo-dGTP. These substrates are similar to the ones being used here. Svensson et al. [24] conjectured that this rate difference was a consequence of the presence of two hydrophobic residues, Leu9 and Val83, in the enzyme structure, preventing any hydrogen bond from being formed between MTH1 and the 2-hydroxyl group of the ribose in the GTP substrate: “The hydroxyl group would, on the contrary, be directed into a hydrophobic pocket composed of Leu9 and Val83, an unfavorable environment for this group.” Both observations suggest that there is a bias in MTH1 that selectively disfavors the binding, and therefore the destruction, of ribonucleotides. This same bias now appears in the energetics of the specificity of the binding site: the presence of the hydrophobic group in Val83 reduces the specificity towards 8-oxo-dGMP, but this loss of specificity is more than offset by the benefit of a reduced energy of binding of ribonucleotides.

H_2_O-2134 contributed significantly towards the specificity. This is corroborated by the hypothesis put forward by Nissink et al. [25], that the increased stabilization of the 8-oxo-dGMP could be attributed to the presence of the nucleophilic hydrogen atom on N_7_ of the substrate, which allowed a cooperative hydrogen bond pair to form. Such a cooperative effect is absent in dGMP, where the water molecule forms two simple hydrogen bonds to O_6_ and N_7_. H_2_O-2134 is also unusual in that the geometric change resulting from replacing 8-oxo-dGMP by dGMP was very large, more than twice the average for the other mutations. This was traced to the effects resulting from a large movement, 1.6 Å, of the hydrogen atom on the water molecule that had originally pointed towards Phe72 when the oxygen atom that had formed a cooperative hydrogen bond with the hydrogen atom on N_7_ on 8-oxo-dGMP was replaced by a normal hydrogen bond in dGMP.

In general, in those regions where the structure was well defined, such as the environment near positions 1, 2, and 6 of the guanine group, the difference in specificity between the two systems was small. Conversely, where a water molecule was present in system A but missing in system B, differences in specificity were large, the main exception to this generalization being Asn33, where the nearby absence of a water molecule in system B had a negligible effect on the specificity, although, as noted earlier, it did have a large effect on the geometry.

## Discussion

The results presented here were obtained using a semiempirical quantum-chemical method. At present, no direct comparison of these results with those of other techniques, particularly experimental methods, is possible; however, indirect evidence of the validity of the methods described here does exist. This evidence can be split into two groups: direct structural comparison with the results of X-ray analysis, and comparison with experimental results and conclusions.

### Structural comparison

All structural features in the region of the binding site were reproduced with good accuracy. In an earlier work [26], the donor–acceptor hydrogen-bond distance between O_δ_ on Asp119 and the indole NH of Trp123 had been predicted to be too short by 0.4 Å. In this investigation, the use of a restraining force [27] that acted on atoms outside the region of the binding site resulted in the RMSD for the binding site decreasing by over 60 %, and the error in the Asp119–Trp123 hydrogen-bond distance decreasing to 0.06 Å.

When unconstrained global optimizations were used, the accuracy of the assignment of energy contributions to the various residues in the binding site was compromised by the residues moving away from their correct positions. To a large degree, this was a result of motion outside the binding site. By using a bias in favor of the PDB geometry to reduce that motion, errors within the binding site were also reduced.

**Fig. 8:**
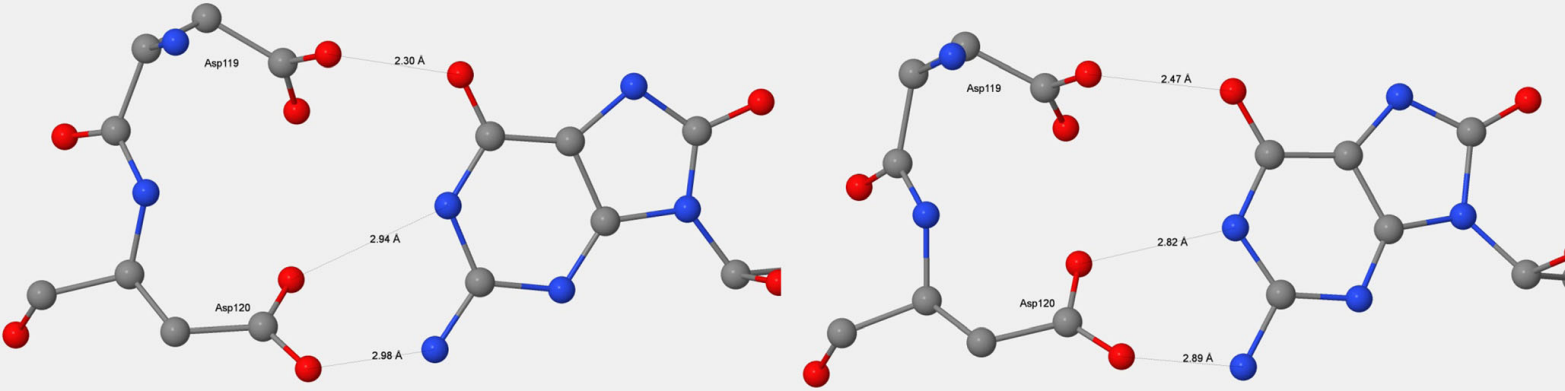
Comparison of the Asp119–Asp120–guanine X-ray and PM7 environments. *Left panel*: structure from chain B in 3ZR0. *Right panel*: structure predicted using PM7

All individual features of interest within the binding site were reproduced. These included the nine-atom Asp–Asp pharmacophore, the short Asp119–guanine donor–acceptor distance (Fig. 8), the Asn33 hydrogen bonds, and the orientation and position of H_2_O-2134.

### Comparison with experiment

#### Binding

With the exception of Met81, the 12 residues and one water molecule in the binding site contributed to the binding of the substrate.

As expected [24, 25, 33], Asp119, Asp120, and Asn33 formed the most stable bonds with the substrate. Of these, the interaction between Asp120 and the guanine was by far the strongest, being about three times that of Asp119. Strong intermolecular interactions that involve two hydrogen bonds of this type have been modeled [34] using high-level methods, which showed that stabilization energies spanned the range from 15 to 19 kcal mol^−1^.

Interestingly, although the O–O distance in the Asp119–guanine hydrogen bond was shorter by about half an Ångstrom than the hydrogen bonds between Asp120 and guanine, the Asp119–guanine binding energy was predicted to be much smaller than that of Asp120–guanine. This is consistent with experimental results. Based on the X-ray structure of 3ZR0, it is incontestable that the geometric effect is real, and, based on mutation experiments, the suggestion has been made [25, 33] that D120 is the key element for binding.

This and related work [26] suggest that both Asp119 and Asp120 are protonated and that the guanine is present as the anion. Both residues were protonated in the optimized PM7 structure, and that structure was similar to the X-ray structure, as shown in Fig. 8. A B3LYP optimization of the solvated acetate anion hydrogen bonding to guanine at H_6_ resulted in the migration of the hydrogen atom from O_6_ to the acetate, giving rise to an acetic acid molecule hydrogen bonding to a guanine anion. All these results suggest that a negatively charged guanine and two neutral Asp residues would be more stable than if the negative charge was located on one or the other of the aspartate residues. This interpretation was further supported by the PM7 prediction that, in the mutant D119A, residue Asp120 would be deprotonated; the absence of a carboxylate group on residue 119 would result in the loss of a strong hydrogen bond that had been stabilized by the negative charge on the guanine, and this loss was sufficient to move the equilibrium towards an ionized Asp120 and a neutral guanine.

#### Specificity

The water molecule and most of the residues in the binding site also contributed to the specificity of the substrate, the exceptions being Val83, Asp120, and Trp123. For Val83 and Asp120, a factor unrelated to the specificity was identified as causing the decrease in specificity. In Val83, the highly hydrophobic environment would selectively disfavor [24] binding by GTP and would therefore reduce the rate at which MTH1 destroyed a useful nucleotide. In the rigid Asp119–Asp120 moiety, the addition of the two specificities weakly favored the binding of the oxidized over the native substrate.

Because of its proximity to the Nudix box, Lys23 would likely have the alternative and possibly more important function of facilitating hydrolysis of the pyrophosphate.

Obviously, residues not near to the binding site should not contribute to the binding or to the specificity. Any significant energy contribution to either process would be evidence of a spurious prediction. When ten such residues were mutated and the geometries reoptimized, the average unsigned energy was 0.09 kcal mol^−1^ for binding and 0.05 kcal mol^−1^ for specificity. That is, no evidence of spurious energy terms was found. To further increase the precision of the specificity calculation, geometry optimization was not used for residues within the binding site; instead, mutation was performed by using pre-optimized rigid residue structures. This resulted in the elimination of all errors of the type caused by the use of finite geometry optimization.

Even after all errors in precision had been eliminated, large differences—up to 1.6 kcal mol^−1^ in the case of Phe72—were present in the specificities of individual residues in going from system A to system B. These differences could not be attributed to errors in atom positions in the PDB structure, as all such random errors would have been eliminated during the initial geometry optimizations. The most important remaining difference between systems A and B involves the presence and location of water molecules. System A in 3ZR0 contained 66 more water molecules than system B, several of which were within the binding site, and two of them, H_2_O-2134 and H_2_O-2024, were predicted to strongly influence the specificity and the binding of the substrate. The fact that there were significant differences in specificity between systems A and B suggested that other water molecules were involved in defining or modifying the location and properties of specific residues.

Since all errors arising from loss of precision were eliminated, the differences in specificity between systems A and B could only be caused by chemical changes in the region of the binding site. In systems A and B, there were eleven and seven water molecules in the region of the binding site, respectively. A comparison of the systems A-8OG, B-8OG, A-GMP, and B-GMP showed that the hydrogen-bonding structure within the binding region was significantly different, and the resulting geometric differences were almost certainly responsible for the differences in specificity.

### Predictions of the locations of water molecules

Of the two entire systems in the asymmetric unit, system B was reported [24] to be the less well defined. The higher quality of system A became apparent during this work when several examples were found where the results obtained for system B were markedly different from those of system A, and, in those instances where the difference was examined, a specific fault was identified in system B. All of the faults that were found and examined involved a water molecule that was present in A but was missing in B.

The hydrogen-bonding network in the region of the binding site showed that every water molecule was ideally positioned to form strong hydrogen bonds with nearby structures. The absence of even one water molecule produced distinct changes that showed up in the form of unexpected results. This was vividly illustrated by the example of H_2_O-2024 in system A. In A-8OG, the optimized geometry reproduced the two hydrogen bonds between Asn33 and N_2_ and N_3_ of the substrate, but in B-8OG, one of these bonds—that between Asn33 and N_3_ of the substrate—was missing, and in its place a hydrogen bond formed between N_δ_ and the deoxyribose ring oxygen. A water molecule, H_2_O-2024, present in A-8OG but absent in B-8OG, formed four hydrogen bonds, including one to the N_δ_ of Asn33 and one to the deoxyribose ring oxygen. In the absence of this specific molecule, Asn33 was able to move nearer to the ring oxygen to form a stronger hydrogen bond than it could with N_3_. That a water molecule was missing in B-8OG was then obvious from the resulting large geometric distortion. When a water molecule was added to B-8OG in the equivalent position to H_2_O-2024 in A-8OG, and the geometry reoptimized, the distortion did not occur.

The missing water molecule in B-8OG had the effect of producing a large geometric distortion in the position of the side-chain carboxamide group in Asn33. In the absence of any other factors that could have produced such a distortion, the presence of this distortion would be indicative of the presence of a water molecule at that location. If the result of adding a water molecule was that the distortion vanished, then the assumption could be made that a water molecule was indeed present but was not resolved in the X-ray analysis.

Other, less easily identifiable, indications of missing water molecules were noticed. In A-8OG, the equivalent of water molecule H_2_O-2134 was not present in B-8OG. This molecule formed strong hydrogen bonds with H_7_ and O_6_ of the substrate in A-8OG, and with O_6_ and N_7_ in A-GMP, and thus contributed to both the binding and the specificity. Adding this molecule to B-8OG increased the similarity to A-8OG without introducing any artefacts. No significant changes occurred in the geometries of the nearby residues or the substrate; the only computational results indicating that a water molecule was missing were the changes in energies. As a result, the de novo detection of a missing water molecule of this type using computational methods might be problematic, but if such a missing molecule were to be suspected, the results of computational simulations could be used as supporting evidence.

Finally, in some cases missing water molecules might be suspected, and anomalies might be detected using computational methods, but no connection between the two could be made. An example of this would be Phe27, which contributed strongly to the specificity in system A but acted in the opposite sense in B, slightly reducing the specificity. Examining the environment of Phe27 did not reveal any reason for this difference. However, in the X-ray structure of 3ZR0, the C_ε2_–C_8_ distances were very different: in A it was 4.00 Å and in B it was 3.37 Å, a difference of 0.63 Å. This difference was not reflected in the calculated structures, where the equivalent distances were 3.90 Å and 3.87 Å. Given the large number of possibilities for the different specificities—different crystal structures for systems A and B, technical difficulties such as disorder, issues arising from the computational models used, etc., any attempt to postulate the presence of one or more missing water molecules would be futile.

### Accuracy

Semiempirical methods have a limited accuracy; in the case of PM7, the average unsigned error (AUE) in Δ*H*_f_ for small organic compounds is 4.1 kcal mol^−1^. Because most systematic errors are removed during the parameterization step of method development, leaving only uncorrelated errors, the AUE should increase as the square root of the size of the system. Most of the systems used here were about 200–300 times larger than those used when validating PM7, so the AUE in the MTH1 systems would be about 60–70 kcal mol^−1^. This would represent the absolute error in Δ*H*_f_. For differences in Δ*H*_f_ of the type used here, a cancellation of errors would occur that would result in a large increase in accuracy. All of the differences in Δ*H*_f_ arise from noncovalent interactions; of these, the largest involve hydrogen bonding and dispersion. In recent years, methods have been developed that increased the accuracy of prediction of these terms to the degree that the error in differences in hydrogen-bond energies due to changes in the system could be regarded as insignificant. Only two errors when determining specificity remain. Specificity in the MTH1 system is undoubtedly due to the electrostatic and electronic effects that result when the atom attached to C_8_ in the substrate changes from hydrogen to oxygen, and to the presence or absence of a hydrogen atom on N_7_. Most of the side chains near to C_8_ are unpolarizable hydrophobic groups, with the exception being Met81, where the methylthio group –S–CH_3_ has a slight polarization [25]. This, presumably, is sufficient to produce its significant specificity in favor of stabilizing the 8-oxo-dGMP substrate. The magnitude of this specificity would depend on how accurately PM7 predicts charge distributions. PM7, like all quantum chemical methods, predicts keto oxygen atoms to have a partial negative charge. The magnitude of this charge would be method-dependent, but it is unlikely to be significantly different from the value of −0.56 given by PM7.

Electronic effects due to the atom attached to C_8_ would propagate to all the atoms in guanine through its π-system. The results obtained in this work suggest that the magnitude of these effects would decrease with increasing distance and be quite small at the 1, 2, and 6 positions, i.e., where the Asp119 and Asp120 residues form hydrogen bonds with guanine.

### Use of high-level methods

Given that semiempirical methods are of limited accuracy, the question arises of how much confidence can be placed in their predictions, particularly those that touch on controversial topics. Examples of these would be the prediction that the position of the hydrogen atom between the carboxylate oxygen of Asp119 and O_6_ of 8OG-1157 would be nearer to the carboxylate, and that Asp120 would be ionized in the D119A mutation. In each such instance, a full geometry optimization was performed using small systems that exemplified the feature in question. In all cases the predictions made by PM7 were in agreement with those of B3LYP.

### Validity of simulations at 0 K

All the computational models used the assumption that the heats of formation at the energy minima were representative of the situation in vivo; that is, that the properties of the system at 0 K could be used as representatives of the same properties at ca. 298 K. This assumption implies that the effects of internal energy at 298 K would not alter the results obtained at 0 K significantly. The alternative—to perform a dynamics calculation on each model and then time-average the results—was considered unnecessary. This conclusion was based on the fact that PM7, like all semiempirical methods, was parameterized to reproduce chemical properties at 298 K; so, by implication, all internal energy phenomena had already been accounted for. Working on the 0 K potential-energy surface would have the added benefit that very small energy differences could be calculated with good precision. For systems of the size described here, simulations run at 298 K would need to run for a long time for the imprecision due to random fluctuations to drop below 0.1 kcal mol^−1^.

### Not facts, but predictions

Computational chemistry can provide various kinds of information. Very high accuracy methods [34–36] can provide results that qualify as reference data for lower level methods. Because of their large computational requirements, these methods are not routinely used for modeling chemistry, but the reference data they generate can be used to parameterize [12, 14, 37–39] and validate other, much faster, methods that can be used for modeling chemistry. Mainstream ab initio methods are fast enough to allow small systems to be modeled routinely, and mixed quantum mechanics–molecular mechanics methods can be used to model large systems, even enzymes. Semiempirical methods are now flexible enough and fast enough that entire enzymes can be modeled easily and quickly. In this work on binding and specificity, the PM7 method in MOPAC2016 has allowed the energy contributions of individual residues to be predicted. The hope is that these results—or, more accurately, predictions—will be of use when attempting to understand phenomena of the type that occur in enzymes.

Philosophical issues arise when computational chemistry predictions are compared with experimental results, particularly when theory and experiment disagree. At its present stage of development, the predictions of semiempirical calculations are obviously of limited value. When such predictions agree with experimental results, it merely confirms the accuracy of the theoretical method. When they disagree, it casts doubt on the validity of the theoretical method. Either way, generating predictions that would be useful would be problematic. Both of these options were avoided in this work by focusing on predicting the individual energy contributions to the specificity of MTH1 for the preferential hydrolysis of oxidized nucleotides, data that could not otherwise be obtained using experimental methods.

Some deductions could be made by examining the X-ray structure. Thus, the importance of hydrogen bonding in the binding site became obvious, but the precise pattern of hydrogen bonding in the Asp119–Asp120 pharmacophore could not be deduced from the X-ray structure because the hydrogen atom positions were not located. The argument might be made that the hydrogen atom positions predicted using PM7 are not facts, and should therefore be regarded as merely conjectures or estimates, and that, of the quantum-theoretical methods, the semiempirical are among the least accurate and thus the least trustworthy. However, good agreement was obtained when comparisons were made with experimental results and with high-level calculations. Therefore, the pattern of hydrogen atoms in the Asp119–Asp120 pharmacophore cannot be easily dismissed; instead, until a better theoretical prediction is made or more factual information becomes available, it should be regarded as representing the best model currently available.

Some results presented here are not supported by either experiment or by high-level calculations. Among these are the most important results, specifically the individual energy contributions. A few of these are qualitatively in agreement with the known properties of the binding site. Others—in particular the large specificity contribution from Phe72 despite it being almost ignored in the literature—were unexpected, neither refuted nor supported by facts, and should therefore be regarded as predictions.

In contrast to experimental work which can take months if not years of effort to generate a single datum, computational simulations are rapid and inexpensive. Most of the results reported here required only one to two minutes to set up and five to ten minutes to run using standard desktop computers, the exception being the initial geometry optimization, which required about seven CPU days of effort.

### Summary of method

This method was designed for use on substrates docked in enzymes that have rigid binding pockets. It is likely that the use of rigid fragments in the prediction of contributions to binding energies might not be suitable for use with enzymes in which large structural changes take place when substrate binding occurs. Because of this, its use should be limited to systems that are similar to the one used here.

For systems of the type used here, the steps involved in calculating the various energy terms are as follows:

- A starting model would be built; this should be the smallest system that would be chemically sensible and would capture all the phenomena of interest. A typical starting point would be the PDB structure to which hydrogen atoms were added. This would be followed by a constrained geometry optimization in which the optimized geometry would be biased towards the original starting geometry. If any large distortions occurred, that would indicate a potential fault in the PDB structure. For example, in the system reported here, the absence of a water molecule near to Asn33 in the B chain resulted in an unexpected motion of the carboxamide group; this error was rectified when a water molecule was added at the appropriate site. All such anomalies should be examined and the system modified so that the distortions are minimized.
- Those parts of the system that interact with the substrate would then be optimized, but without the constraint being used and with all other atoms held fixed. This would result in an optimum structure: the substrate and binding site would be in equilibrium with the rest of the system, and the rest of the system would be biased towards the original PDB structure. When specificity is of interest, this step would need to be performed for each candidate substrate.
- Individual residues in the binding pocket would then be mutated, one at a time, to remove the groups that interact with the substrate, and the geometries of the mutated residues reoptimized.
- All energies of interest would be calculated using rigid fragments. Thus, to determine the energy contribution of binding arising from a specific side chain, the heat of formation of the unmutated system would be compared with that of the system in which the rigid residue was replaced by the rigid mutated residue, for the enzyme both with and without the substrate docked in the binding site. To predict specificity, the system consisting of the enzyme without the substrate would be replaced with the enzyme with the other substrate docked in the binding site. Because very high precision is required in all these calculations, rigid fragments would need to be used in order to eliminate errors of the type that result from a geometry optimization calculation.

## Conclusions

Using current computational semiempirical quantum chemistry methods, individual energy contributions to various processes in enzyme chemistry can be modeled with useful accuracy. These energies were obtained by modeling the effects of changing individual residues so that the mutated residue did not interact significantly with the substrate. Using the oxidized nucleotide pyrophosphohydrolase enzyme MTH1, individual contributions to the binding energy attributable to each residue in an active site were calculated using PM7, and the results were in good agreement with both experimental and X-ray analysis reports. The interesting binding site Asp119–Asp120 was examined and an internally consistent description was developed of the structural and energy changes that occur when docking takes place.

Of the 11 residues in the binding site, Asp119, Asp120, and Asn33 were the most important contributors to the energy of stabilization of the substrate, and Phe72, Asn33, Met81, and Trp117 were the most important contributors to the energy of specificity of MTH1 for 8-oxo-dGMP over dGMP. A water molecule located in an amphipathic pocket in the binding site formed hydrogen bonds with O_6_ and N_7_ of dGMP and with O_6_ and H_7_ of 8-oxo-dGMP, and contributed significantly to both the stabilization and the specificity. One residue, Val83, was predicted to make only a small contribution to the stabilization and a negligible contribution to the specificity energy. On the other hand, this specific residue had earlier been identified as being responsible for reducing the catalytic activity of MTH1 to hydrolyze other, beneficial, nucleotides, and the advantage resulting from not destroying beneficial nucleotides presumably outweighed the penalty of reduced specificity. All these results were in accordance with experimental observations.

During the simulations, some unrealistic results were obtained that were traced back to reported faults in the original PDB structure. The quality of the results was improved when two of these faults were corrected. Reversing this sequence suggests a method for improving the computational model: when unrealistic results are obtained, these could be used as an indicator of where water molecules should be added to the starting structure to make it more realistic.

Provided care is taken to minimize both modeling and computational errors, very low-energy quantities—such as the individual energy contributions of each residue that influence specificity—can be calculated. Computational methods are philosophically different from experimental methods in that they cannot provide facts about nature; they can only provide predictions. Nevertheless, when sufficient care is taken, these predictions can provide a useful new perspective for viewing chemical phenomena. The methods described here for calculating individual residue contributions to the energies of binding and specificity are not specific to any one enzyme, and hopefully will prove useful when investigating other enzyme systems.
